# CTHRC1 is a prognosis-related biomarker correlated with immune infiltrates in colon adenocarcinoma

**DOI:** 10.1186/s12957-022-02557-7

**Published:** 2022-03-20

**Authors:** Chuang Meng, Yue Zhang, Dujun Jiang, Jian Wang

**Affiliations:** 1 The First Clinical Medical College, Xuzhou Medical University, Xuzhou, 221004 Jiangsu China; 2 Department of Gastrointestinal Surgery, Xuzhou Cancer Hospital, Xuzhou, 221005 Jiangsu China; 3 Department of General Surgery, General Hospital of Xuzhou Mining Group, Xuzhou, 221006 Jiangsu China

**Keywords:** CTHRC1, COAD, Prognosis, Biomarker, Immune infiltration

## Abstract

**Background:**

Colon adenocarcinoma (COAD) is one of the common cancers worldwide. Collagen triple helix repeat containing 1 (CTHRC1) has been reported to be involved in cell invasion, angiogenesis, and the promotion of epithelial-mesenchymal transformation by mediating multiple signaling pathways. However, the role of CTHRC1 in COAD has not yet been determined.

**Methods:**

Differentially expressed genes were evaluated using gene expression data from the Oncomine and TIMER databases. Correlations between CTHRC1 gene expression and clinicopathological factors were analyzed using gene expression data from UALCAN databases. Then, we searched the GEPIA database to evaluate the association of CTHRC1 gene expression with clinical outcomes. The cBioPortal database was used to analyze CTHRC1 genetic alterations. Subsequently, the TIMER website was chosen to assess the correlation of CTHRC1 with the tumor immune cell infiltration level. The TCGA dataset was used for a gene set enrichment analysis (GSEA).

**Result:**

CTHRC1 was highly expressed in COAD patients, and significantly related to poor prognosis. In addition, elevated expression of CTHRC1 was related to the clinical stage and pathological type of COAD. The GSEA analysis showed that CTHRC1 was enriched in Gα signaling, GCPR ligand binding, neutrophil degranulation, interleukin signaling, and tumor-associated pathways. In addition, CTHRC1 was significantly associated with the expression of multiple immune markers related to specific immune cells.

**Conclusion:**

This study suggest that CTHRC1 expression is related to the prognosis and immune infiltration of COAD patients. Therefore, CTHRC1 may be a new candidate prognostic biomarker for determining immune infiltration levels and providing COAD prognoses.

## Introduction

Colon adenocarcinoma (COAD) is a common malignant cancer and the second most common cause of cancer-related deaths worldwide [1]. The internal epithelial cells of colorectal tissues are derived from endodermal cells, and a multi-step process occurs from normal epithelium to adenomatous polyps to invasive colorectal cancer, which is achieved by inactivating tumor suppressor genes and activating oncogenes [2]. The treatment response and survival rate of patients with advanced COAD are still very low compared to the early stage of COAD, with 5-year survival rates dropping from 50 to 10% in more advanced cases. Surgical tumor resection is the mainstay treatment of radical treatment for locally advanced COAD, while a treatment strategy is not available for metastatic tumors that cannot be surgically resected or have poor efficacy of chemotherapy and radiotherapy [3]. Quantitative analyses of COAD indicate that stem cells are initially transformed into malignant tumor cells over a 10-year period, and then these tumor cells acquire the ability to metastasize over the next 5 years [4]. Early and accurate detection of COAD is important for disease control and prevention because approximately 30–40% of COAD patients relapse after treatment [5]. Given the clinical challenges of the effectiveness of therapeutic strategies to improve the outcome of COAD patients, a significant proportion of patients receiving conventional treatment still experience relapse. Therefore, it is imperative to find new methods, especially molecular markers, to perform timely diagnosis and assessments of colorectal cancer, identify new drug targets, and to better understand the basic molecular mechanisms of COAD for the successful development of effective treatment.

Many studies have reported that the abnormal expression of many genes in tumor tissues may increase the risk of cancer [6]. Therefore, the identification of changes in gene expression levels will provide new insights for studying the pathogenesis of COAD. The collagen triple helix repeat containing 1 (CTHRC1) gene is located on human chromosome 8q22.3 and was initially identified in balloon injury and normal rat arteries [7]. Its overexpression in fibroblasts is related to an increase in cell migration, motility and invasion [8]. In adults, CTHRC1 is expressed only in the bone matrix and periosteum. CTHRC1 is also present in atherosclerotic plaques and mineralized bone matrix [8]. In other tissues, CTHRC1 is expressed at sites that significantly overlap with interstitial collagen and members of the transforming growth factor-β (TGF-β) family, particularly bone morphogenetic proteins (BMPs) [9]. In addition, the expression of CTHRC1 was positively correlated with lymph node metastasis, tumor stage, and disease prognosis. CTHRC1 is widely upregulated in several solid tumors, including melanoma, gastrointestinal cancer, breast cancer, thyroid cancer, liver cancer, and pancreatic cancer [10]. Recent studies have shown that CTHRC1 can trigger tumor metastasis by promoting epithelial-mesenchymal transformation, promoting cell invasion, and inducing angiogenesis through a variety of signaling pathways [11]. Although the high expression of CTHRC1 and the prognostic value of COAD patients have been highlighted by previous studies, the predictive accuracy of CTHRC1 as a biomarker can vary widely in heterogeneous diseases such as COAD due to the lack of adequate sample sizes and stringent inclusion criteria.

Dysregulation of CTHRC1 expression in COAD and its relationship with clinical pathological features and prognosis have been partially reported. However, bioinformatics analysis has not been applied to explore the role of CTHRC1 in COAD. With the development of RNA-seq technology, genome sequencing has experienced huge changes. In this study, the Cancer Genome Atlas (TCGA) and Oncomine database were employed to investigate the expression and function of CTHRC1 in COAD. The relationship between CTHRC1 levels and clinical pathological parameters was analyzed, and CTHRC1 was identified as a potential biomarker of tumor progression for COAD. Furthermore, we analyzed mutations of CTHRC1 in COAD to determine its expression pattern, potential function, and prognostic value.

## Materials and methods

### TCGA database

TCGA (www.tcga-data.nci.nih.gov/tcga/) contains more than 10,000 samples of 39 tumor types (16). The TCGA-COAD dataset was acquired from UCSC Xena database (http://xena.ucsc.edu/), and it is processed uniformly by the TOIL process, which is free of computational batch effects [12].

### Oncomine database analysis

Oncomine (http://www.oncomine.org) is a gene chip-based database for gene expression analysis, coexpression analysis, enrichment analysis, and interaction network analysis in various cancers [13]. It contains 715 datasets and 86,733 samples. This database uses Student’s *t* test to compare the transcription levels of CTHRC1 in normal controls and clinical cancer specimens. The *p* value was set as 0.05 and the fold change was set as 1.5.

### UALCAN database analysis

The UALCAN database (http://ualcan.path.uab.edu/) is available for online analysis of differential gene expression in cancer and normal tissue from TCGA and MET500 data [14]. This study used the UALCAN database to determine the correlation between CTHRC1 gene expression and various sub-groups of clinical characteristics. *p* < 0.05 was considered statistically significant.

### GEPIA database analysis

GEPIA (http://gepia.cancer-pku.cn) is a newly developed interactive web server for gene expression analysis based on TCGA and GTEx data [15]. It includes 9736 tumor and 8587 normal samples. In the current study, survival analysis of CTHRC1 was evaluated using TCGA-COAD datasets with a median cut-off. Kaplan–Meier (KM) plots are presented with hazard ratios (HRs), 95% confidence intervals (CIs), and log-rank *p* values.

### PrognoScan database analysis

The correlation between CTHRC1 expression and COAD survival was also analyzed by the PrognoScan database (http://www.abren.net/PrognoScan/) [16]. To select the datasets to be included in this study, the screening parameters were set as follows: “Cancer Type” as COAD, “Gene” as CTHRC1. The HR with 95% CIs was calculated. The threshold was adjusted to a Cox *p* value < 0.05.

### TISIDB database analysis

TISIDB (http://cis.hku.hk/TISIDB/index.php) is a web portal for tumor and immune system interactions that integrates multiple heterogeneous data types [17]. It contains genomics and transcriptomics of 30 cancer types from TCGA, RNA sequencing data set of patient cohorts treated with immunotherapy. It was used to investigate correlations between CTHRC1 and different immune genes.

### Tumor-infiltrating immune cell analysis

Tumor-infiltrating immune cell analysis (TIMER) (http://www.timer.cistrome.org/) is a website used to analyze the expression of various types of cancer-associated genes and tumor-infiltrating immune cells [18]. It integrates 10,897 samples across 32 types of cancer in the TCGA. The site provides estimates of immune invasion abundance through multiple immunodeconvolution methods and allows users to dynamically generate high-quality numbers to comprehensively explore tumor immunological, clinical, and genomic characteristics. In this study, the correlations of CTHRC1 expression with immune infiltration in COAD were evaluated by purity-correlated partial Spearman’s correlation values and the statistical significance.

### cBioPortal database analysis

cBioPortal (http://www.cbioportal.org) is an online access database used to explore cancer genomic data from multiple perspectives [19]. The gene mutation and survival data derive from 640 COAD samples in the TCGA database in cBioPortal. The genome atlas includes mutations and putative copy number changes in the genome identification of important cancer targets. OncoPrint was constructed to directly reflect all types of changes in CTHRC1 gene amplification, deletion, mRNA upregulation, and mRNA downregulation in COAD patients. In addition, the overall survival (OS) and disease-free survival (DFS) of CTHRC1 gene alteration were analyzed through the “Comparison/Survival” module in cBioPortal.

### Gene–gene interaction and protein–protein interaction networks (GeneMANIA)

GeneMANIA (http://www.genemania.org) can be used to generate hypotheses about gene functions, analyze gene lists, and preferentially select genes for functional analysis [20]. A protein-protein interaction network between CTHRC1 and its 50 adjacent genes was constructed by GeneMANIA. Then, these 50 genes were analyzed by gene ontology (GO). GO enrichment analysis predicts the functional role of target host genes based on three aspects, including molecular function, biological processes, and cellular components.

#### Gene set enrichment analysis

Gene set enrichment analysis (GSEA) is a calculation method based on the entire gene expression matrix and an analysis method that determines whether a previously defined set of genes shows statistically significant and consistent differences between two phenotypes. In this study, ClusterProfiler 3.11 was used to analyze the TCGA-COAD dataset to elucidate significant functional and signaling pathway differences between the high and low CTHRC1 groups. The CTHRC1 gene expression level was used as a phenotypic marker, and each analysis carried out 1000 permutations of gene sets. In this study, H.all.v7.0.symbols.gmt from MSigDB Collections was selected as the reference gene set. The statistically significant GSEA threshold was set as *p* < 0.05 and FDR < 0.25. The enrichment of phenotypic was sequenced using calibrated *p* values and normalized enrichment scores.

### Statistical analysis

CTHRC1 expression was analyzed via the Oncomine, TIMER, and UALCAN databases. The results of PrognoScan and GEPIA are displayed with HR and P or Cox *p* values from a log-rank test. Spearman’s correlation analysis was performed to evaluate the correlation of gene expression in TIMER. The GO and GSEA analysis were performed by “ClusterProfiler” R package. All R packages were operated using R software version v3.6.3. *p* values < 0.05 were considered statistically significant.

## Result

### Expression of CTHRC1 in different cancers

**Fig. 1:**
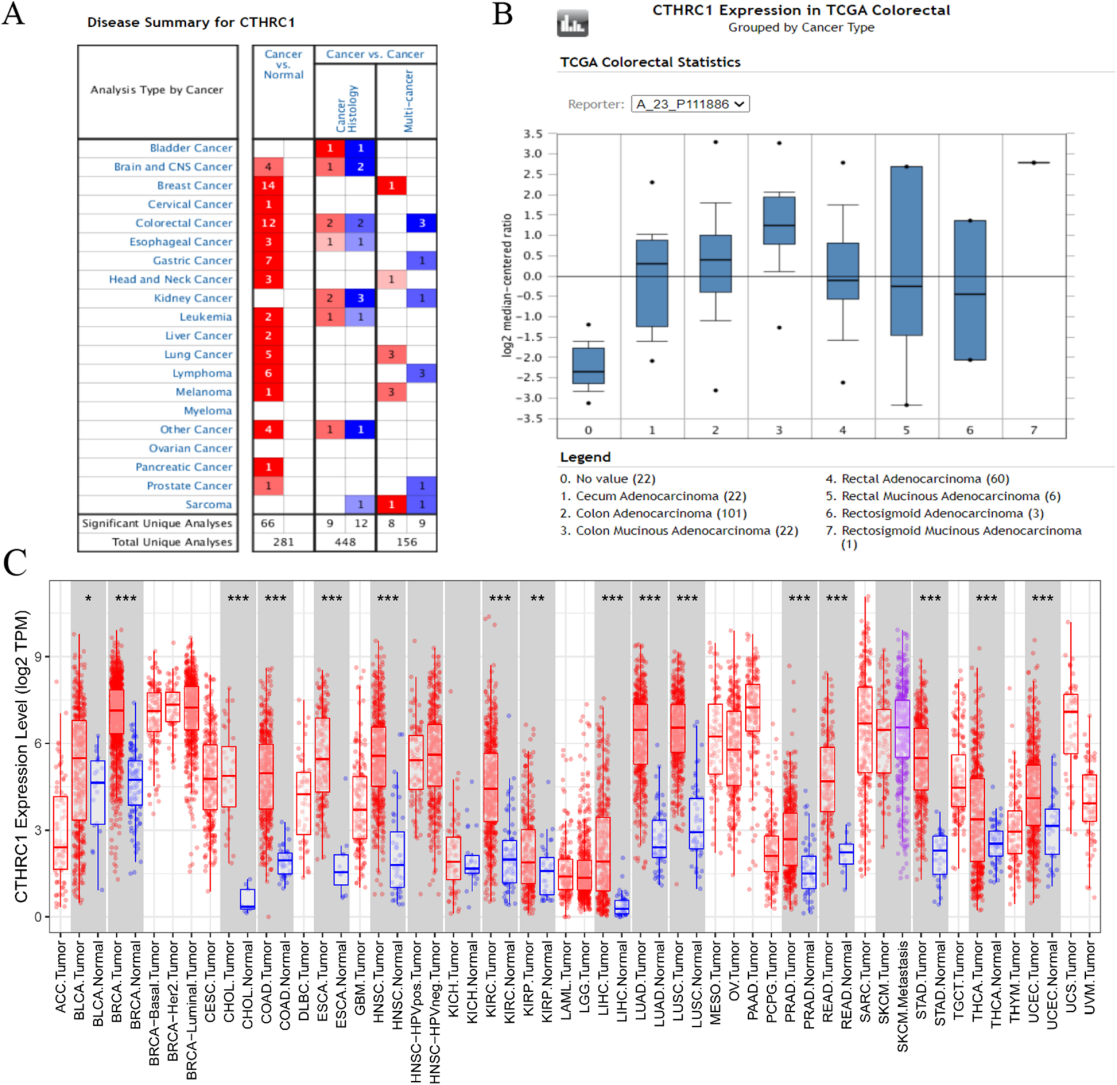
Expression analysis of CTHRC1 by Oncomine and TIMER databases. **A** Expression of CTHRC1 in different types of human cancers in the Oncomine database. **B** CTHRC1 is over-expression in different types of intestinal tumor. **C** Expression of CTHRC1 in different types of human cancers in the TIMER database

**Fig. 2:**
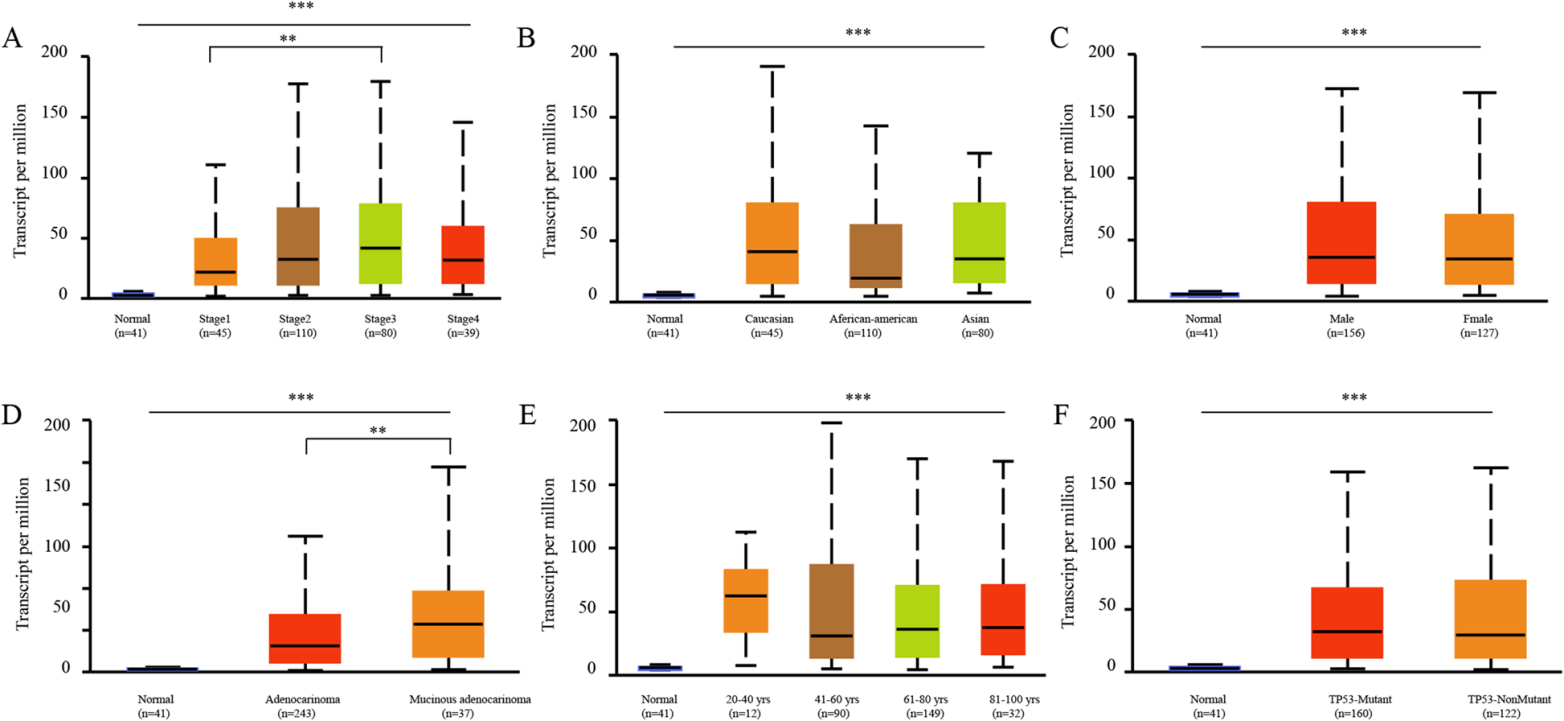
Box plot revealing the relationship between CTHRC1 expression and different clinical indicators. **A** Clinical stages, **B** race, **C** gender, **D** histological status, **E** age, **F** TP53-mutant. **p < 0.01, ***p < 0.001

Oncomine and TIMER were used to analyze the expression of CTHRC1 mRNA in COAD and normal tissues. The results showed that CTHRC1 mRNA levels in various cancer tissues were significantly higher than those in normal tissues (Fig. 1A). In addition, CTHRC1 mRNA was significantly upregulated in all intestinal tumors (Fig. 1B). Then, we further assessed these data through the TIMER database and found that CTHRC1 mRNA expression was significantly elevated in most tumors compared with normal tissues, especially in COAD (Fig. 1C). According to TCGA data, CTHRC1 expression was 6.222-fold higher in colorectal adenocarcinoma, 10.224-fold higher in mucinous colon adenocarcinoma, 4.803-fold higher in rectal adenocarcinoma, and 4.643-fold higher in cecum adenocarcinoma compared with normal tissues (Table 1). Then, we evaluated the correlation between the expression of CTHRC1 and the clinicopathological features (age, gender, race, clinical stage, histological, and TP53 mutation status) of COAD through the online cancer OMICS database of UALCAN. According to age, gender, race, clinical stage, histological, and TP53 mutation status, CTHRC1 expression was significantly upregulated in COAD patients compared to the corresponding normal controls (Fig. 2). In COAD patients, CTHRC1 expression levels were positively associated with clinical stages and histological status. The expression of CTHRC1 in stage 3 was higher than in stage 1 (*p* < 0.05). The expression of CTHRC1 in mucinous adenocarcinoma was higher than in adenocarcinoma (*p* < 0.05)

**Table 1:** The significant changes of CTHRC1 expression in transcription level between different types of intestinal tumor and normal intestinal tissues (Oncomine database)

	Types of CRC vs. normal	Fold change	***p*** value	t test	Source
CTHRC1	Colon Adenocarcinoma vs. Normal	6.222	1.66E-29	17.582	TCGA
	Colon Mucinous Adenocarcinoma vs. Normal	10.224	4.01E-15	13.761	TCGA
	Rectal Adenocarcinoma vs. Normal	4.803	6.58E-20	12.027	TCGA
	Cecum Adenocarcinoma vs. Normal	4.643	3.89E-9	8.174	TCGA

### High expression of CTHRC1 was related to poor prognosis in COAD patients

**Fig. 3:**
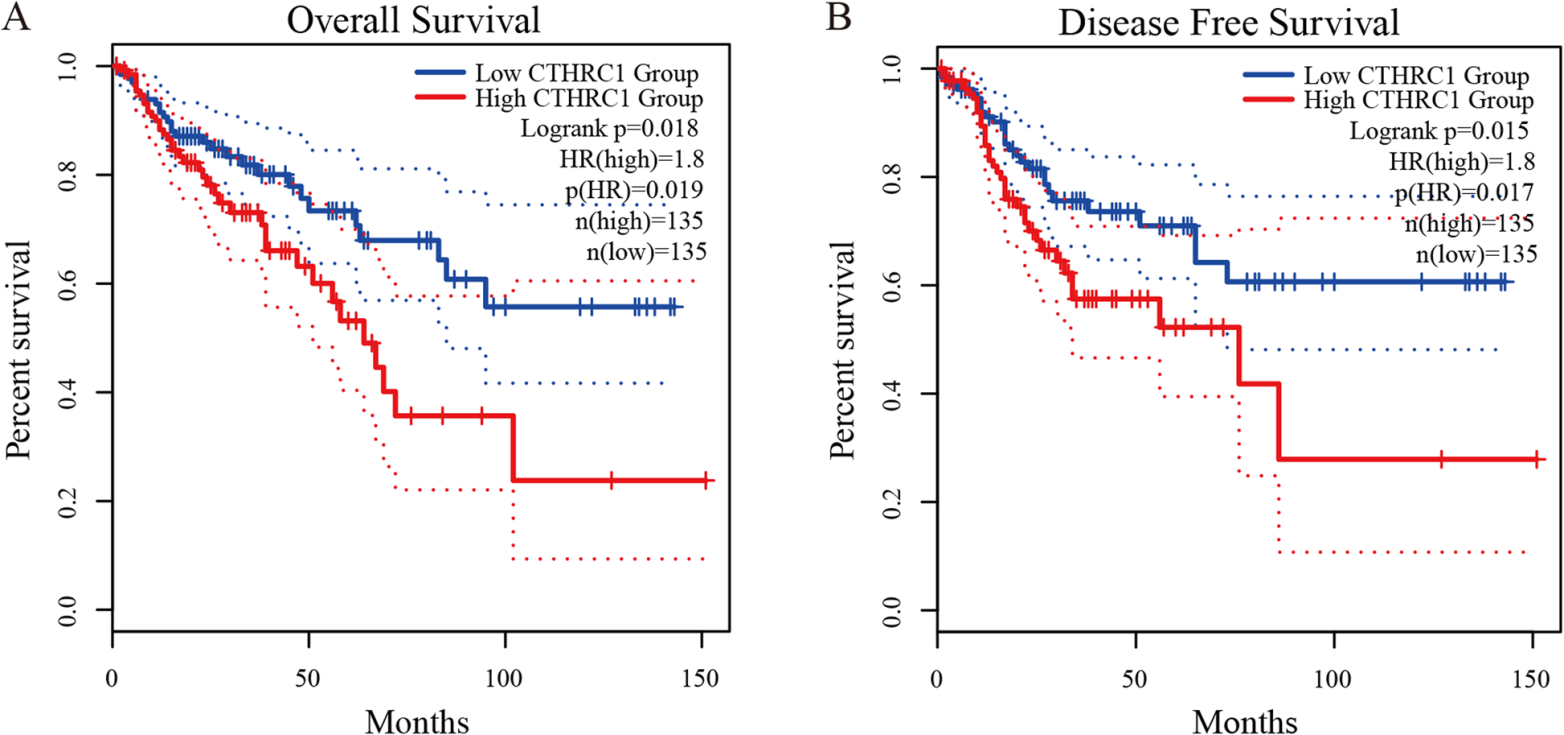
Prognostic value of the CTHRC1 expression in COAD. Survival curves using the Kaplan–Meier plots are shown for OS (**A**), PFS (**B**). *HR* hazard ratio

We further explored the critical efficiency of CTHRC1 in the survival of patients with COAD by using Kaplan–Meier Plotter. The Kaplan–Meier curve and log-rank test analyses revealed that increased CTHRC1 mRNA levels were significantly correlated with overall survival (OS) and disease-free survival (DFS) (Fig. 3A, B). COAD patients with high CTHRC1 expression had poor prognosis (OS, HR = 1.8, *p* = 0.018; DFS, HR = 1.8, *p* = 0.015). Moreover, the PrognoScan Database showed that overexpression of CTHRC1 was significantly related to low OS, DFS, and disease-specific survival (DSS) (Table 2).

**Table 2:** CTHRC1 expression and survival data of COAD patients using the PrognoScan database

Dataset	End point	Probe ID	N	Corrected ***p*** value	HR (95CI%)
GSE17536	Disease Free Survival	225681_at	145	0.007267	1.82 [1.24–2.67]
GSE17536	Overall Survival	225681_at	177	0.001407	1.67 [1.23–2.26]
GSE17536	Disease Specific Survival	225681_at	226	0.012180	1.53 [1.17–2.01]

### Genetic alterations of CTHRC1 in COAD

**Fig. 4:**
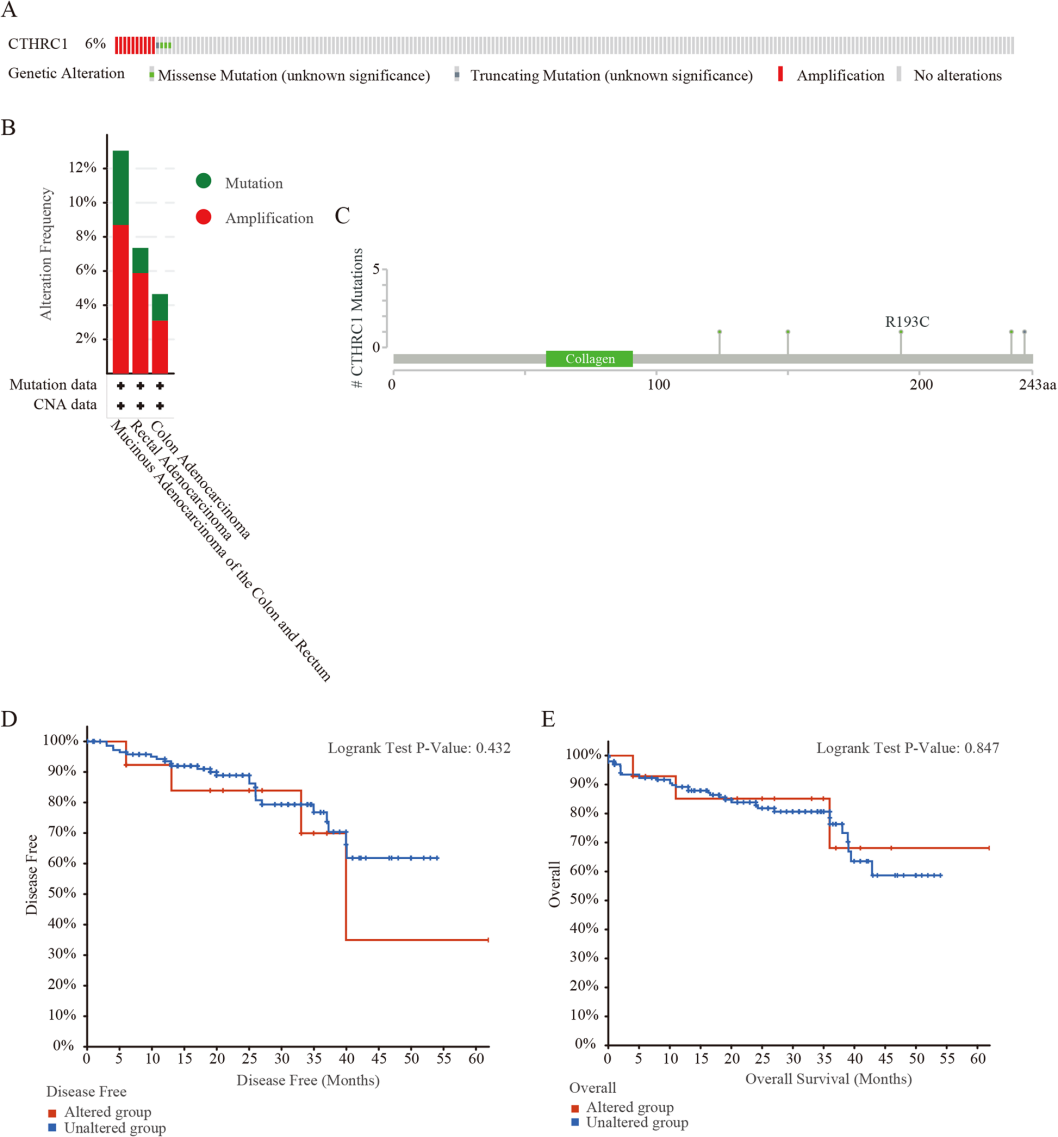
CTHRC1 gene expression and mutation analysis in colorectal adenocarcinoma (cBioPortal). **A** The mutations in CTHRC1 in colorectal adenocarcinoma tissues were shown from top to bottom. **B** Frequency of gene alterations in CTHRC1 in different types of colorectal adenocarcinoma. **C** The details of all mutations in colorectal adenocarcinoma. **D** Kaplan–Meier Plots are used to analyze the correlation between gene alterations in CTHRC1 and DFS, as well as the OS **E** of colorectal adenocarcinoma patients

**Fig. 5:**
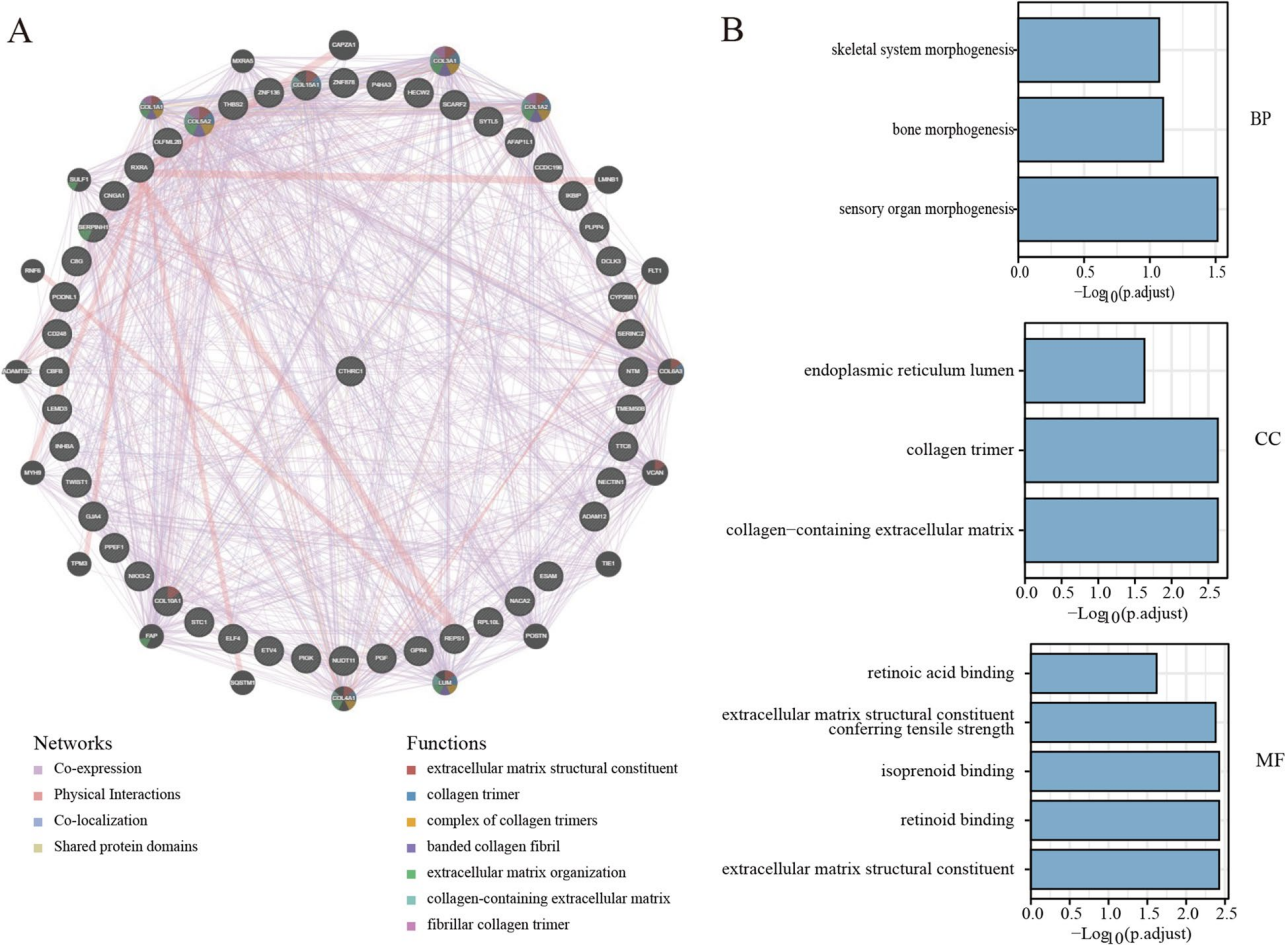
Gene regulation network. **A** The network for CTHRC1 and the 50 most frequently altered neighbor genes. **B** GO enrichment analysis predicted the functional roles of target host genes based on three aspects, including **A** biological processes, **B** cellular components, and **C** molecular functions

Gene alterations in CTHRC1 were found to occur in 6% of the 220 colorectal adenocarcinoma cases in the data obtained from the OncoPrint schematic of cBioPortal. The mutation rate of COAD was 4.56%. Meanwhile, missense mutations and amplification were the main alteration types in mucinous adenocarcinoma of the colon and rectum, rectal adenocarcinoma and colon adenocarcinoma (Fig. 4A, B). Figure 4C summarizes the details of all mutations: CTHRC1 has one truncation mutation and four missense mutations. In addition, we assessed the association of CTHRC1 gene alterations with survival in patients with colorectal adenocarcinoma. However, the OS and DFS were not associated with changes in the CTHRC1 gene in colorectal adenocarcinoma patients. We then constructed the protein-protein interactions network for CTHRC1 and the 50 altered neighboring genes by using GeneMANIA. The results showed that ELF4, PGF, IKBIP, SERPINH1, ESAM, COL15A1, THBS2, COL5A2, COL10A1, ADAM12, OLFML2B, GJA4, CD248, INHBA, PLPP4, HECW2, P4HA3, COL3A1, COL1A2, FLT1, COL6A3, VCNA, TIE1, LUM, COL4A1, FAP, MYH9, ADAMTS2, COL1A1, MXRA5, and SULF1 genes were closely related to CTHRC1 alterations (Fig. 5A). The functions of CTHRC1 and the genes significantly associated with CTHRC1 alterations were predicted by analyzing GO. GO enrichment analysis predicts the functional role of target host genes from three aspects: biological process, cell composition, and molecular function. The results showed that morphogenesis, collagen trimer, endoplasmic reticulum lumen, and other processes were controlled by changes in CTHRC1 (Fig. 5B) (Table 3). The GO enrichment analysis comprised three aspects: a biological process (BP), a molecular function (MF), and a cellular component (CC). The results showed that morphogenesis, collagen trimer, endoplasmic reticulum lumen, and other processes were significantly regulated by the CTHRC1 alterations (Fig. 5B) (Table 3).

**Table 3:** Results of GO analysis

ONTOLOGY	ID	Description	GeneRatio	BgRatio	***p*** value	***p***.adjust	***q*** value
BP	GO:0090596	Sensory organ morphogenesis	6/44	256/18670	2.86e-05	0.031	0.026
BP	GO:0060349	Bone morphogenesis	4/44	114/18670	1.48e-04	0.079	0.068
BP	GO:0048705	Skeletal system morphogenesis	5/44	239/18670	2.38e-04	0.085	0.073
CC	GO:0062023	Collagen-containing extracellular matrix	7/47	406/19717	4.61e-05	0.002	0.002
CC	GO:0005581	Collagen trimer	4/47	87/19717	5.46e-05	0.002	0.002
CC	GO:0005788	Endoplasmic reticulum lumen	5/47	309/19717	8.19e-04	0.023	0.019
MF	GO:0005201	Extracellular matrix structural constituent	5/43	163/17697	4.52e-05	0.004	0.003
MF	GO:0005501	Retinoid binding	3/43	35/17697	8.28e-05	0.004	0.003
MF	GO:0019840	Isoprenoid binding	3/43	36/17697	9.02e-05	0.004	0.003
MF	GO:0030020	Extracellular matrix structural constituent conferring tensile strength	3/43	41/17697	1.34e-04	0.004	0.003
MF	GO:0001972	Retinoic acid binding	2/43	19/17697	9.61e-04	0.024	0.019

*BP* biological processes, *CC* cellular components, *MF* molecular functions

### GSEA identifies signaling pathways associated with CTHRC1

**Fig. 6:**
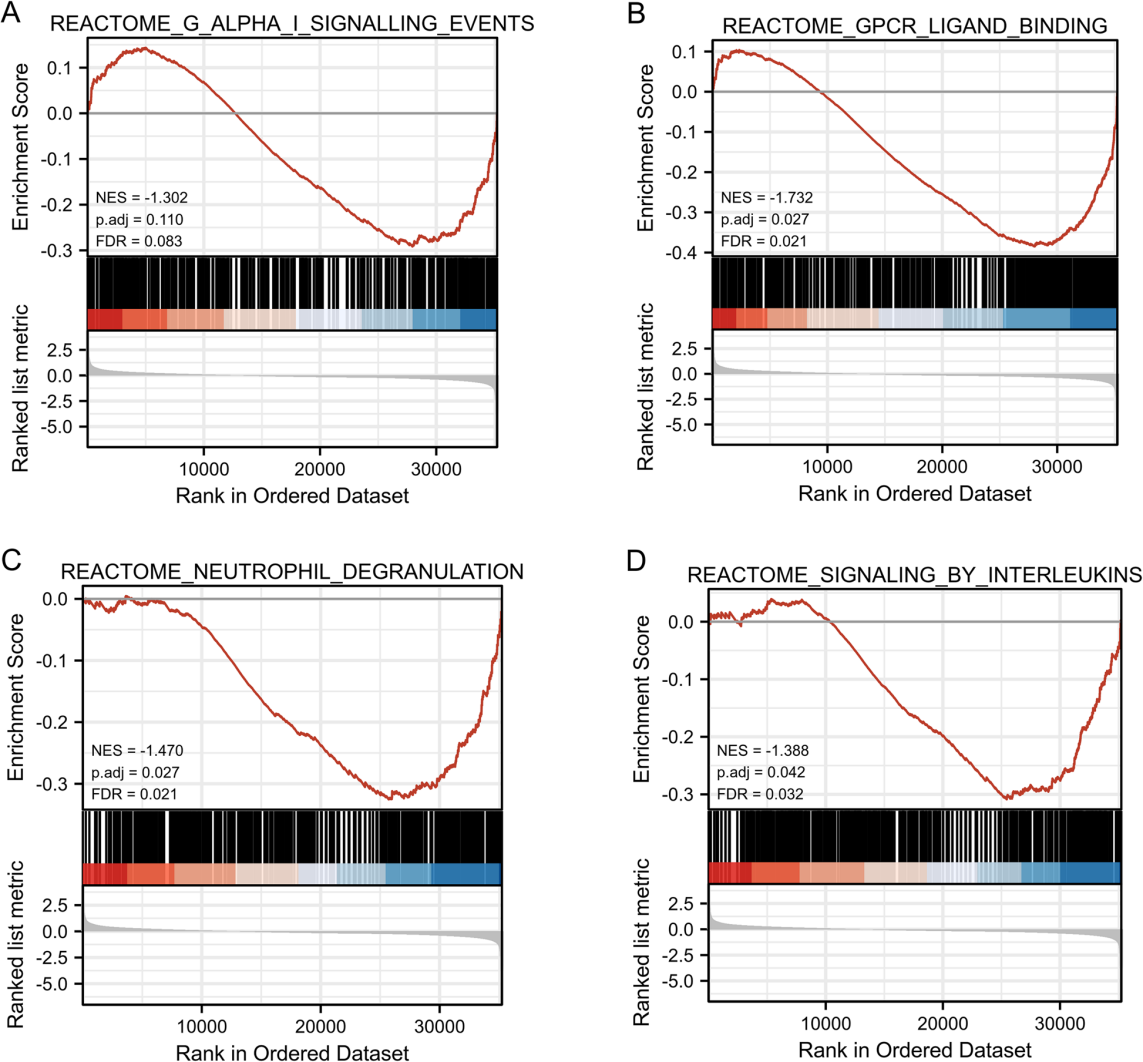
Enrichment plots from GSEA. Gene set enrichment plots of **A** G Alpha I, **B** GPCR ligand binding, **C** neutrophil degranulation, **D** signaling by interleukins in COAD cases with high CTHRC1 expression. RNA sequence data from 480 patients with COAD and 41 normal tissues

We acquired the CTHRC1 expression dataset from the TCGA-COAD data (480 patients with COAD and 41 normal tissues) for the GSEA analysis to identify different activated signaling pathways in COAD. Gene sets associated with Gα signaling, GPCR ligand binding, neutrophil degranulation, interleukin signaling, and tumor-associated pathways showed varying degrees of enrichment in the highly expressed phenotype of the CTHRC1 gene (Fig. 6) (Table 4).

**Table 4:** Results of GSEA

Description	setSize	Enrichment score	NES	***p*** value	***p***.adjust	***q*** values
REACTOME_G_ALPHA_I_SIGNALLING_EVENTS	401	0.535	1.560	0.001	0.015	0.010
REACTOME_GPCR_LIGAND_BINDING	459	0.608	1.774	0.001	0.015	0.010
REACTOME_NEUTROPHIL_DEGRANULATION	474	0.547	1.600	0.001	0.015	0.010
REACTOME_SIGNALING_BY_INTERLEUKINS	458	0.543	1.586	0.001	0.015	0.010
KEGG_PATHWAYS_IN_CANCER	325	0.503	1.459	0.001	0.015	0.010
NABA_SECRETED_FACTORS	342	0.597	1.735	0.001	0.015	0.010
REACTOME_CLASS_A_1_RHODOPSIN_LIKE_RECEPTORS_	327	0.643	1.867	0.001	0.015	0.010
REACTOME_EXTRACELLULAR_MATRIX_ORGANIZATION	300	0.713	2.068	0.001	0.015	0.010
REACTOME_LEISHMANIA_INFECTION	306	0.623	1.808	0.001	0.014	0.010
WP_FOCAL_ADHESIONPI3KAKTMTORSIGNALING_PATHWAY	303	0.590	1.711	0.001	0.014	0.010

*NES* normalized enrichment score

### Relationship between CTHRC1 expression and immune checkpoint genes

**Fig. 7:**
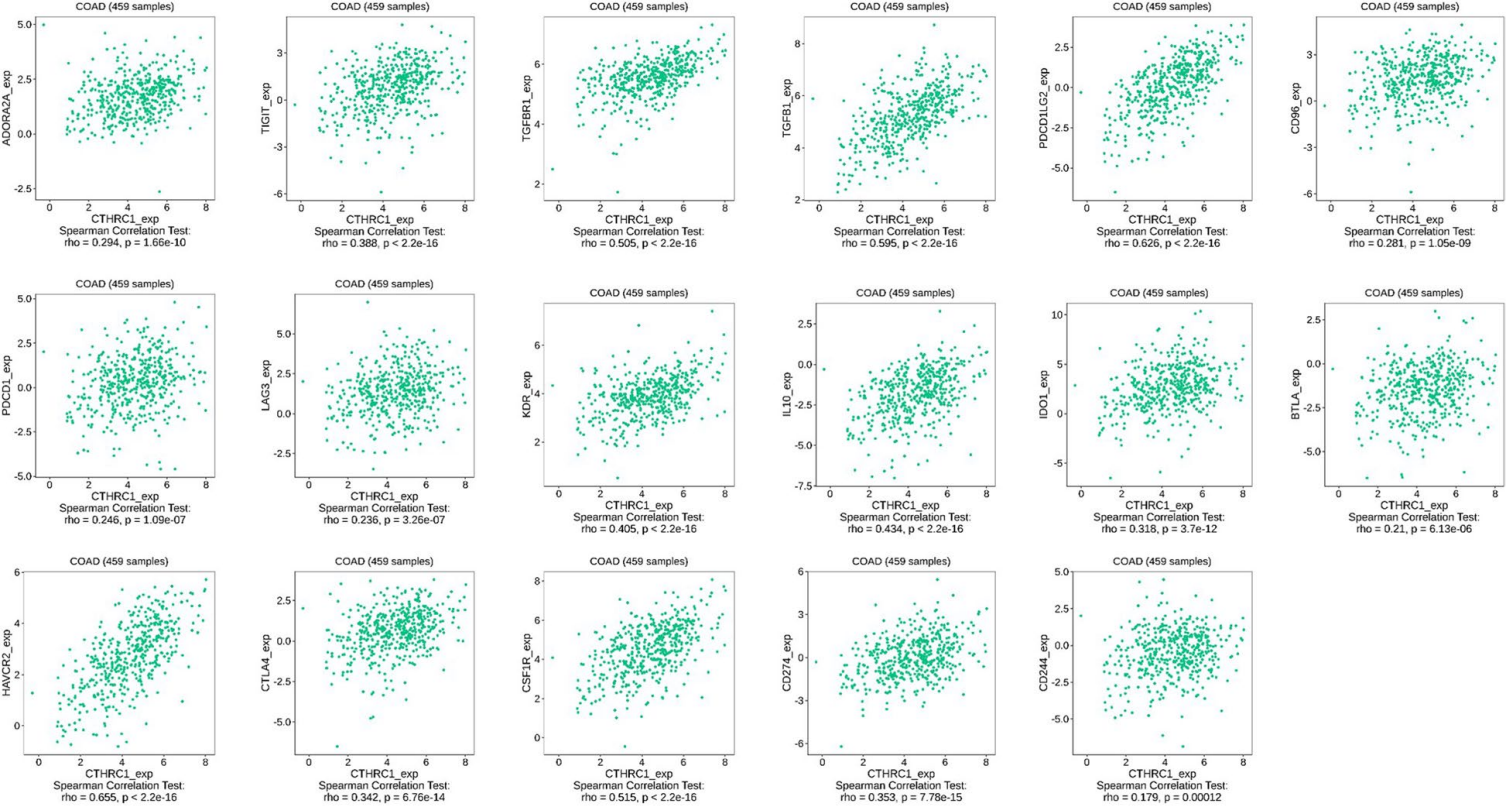
Correlations of CTHRC1 expression and expression of different immune genes in COAD

With increased awareness of immune checkpoint function in humans, immune checkpoint inhibitors have made great progress in cancer treatment. We assessed whether the expression of CTHRC1 was associated with immune checkpoint genes. We used the TISIDB database to study the relationship between CTHRC1 expression and immunosuppressive effects. The results showed that CTHRC1 was associated with ADORA2A, TIGT, TGFBR1, TGFB1, PDCD1LG2, CD96, PDCD1, LAG3, KDR, IL10, IDO1, BTLA, HAVCR2, CTLA4, CSF1R, CD274, and CD244 (Fig. 7).

### Relationship between CTHRC1 expression and immune infiltration

**Fig. 8:**
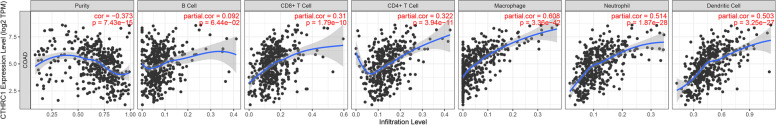
Correlation of CTHRC1 expression with immune infiltration levels in COAD

In patients with cancer, the level of immune cells is closely related to the proliferation and development of cancer cells. Then, we used the TIMER database to explore the relationship between CTHRC1 expression and the degree of immune cell infiltration. CTHRC1 expression was positively correlated with CD4+T cell infiltration, CD8+T cell infiltration, macrophage infiltration, neutrophil infiltration, and dendritic cell infiltration, expect at the B cell level (Fig. 8). To further explore the relationship between CTHRC1 expression in COAD and immune cell invasion level, we identified the correlation between CTHRC1 expression and various immune invasion-associated biomarkers. Our results showed that CTHRC1 expression was significantly correlated with most B cells, T cells, CD8+T cells, macrophages, neutrophils, dendritic cells, and NK cell markers (Table 5).

**Table 5:** Correlation analysis between CTHRC1 and relate genes and markers of immune cells in TIMER

Description	Gen markers	COAD			
		None		Purity	
		Cor	***p***	Cor	***p***
**B cell**	CD19	0.204084	1.07E-05	0.204084	1.07E-05
	CD79A	0.285476	4.89E-10	0.285476	4.89E-10
**T cell (general)**	CD3D	0.292765	1.67E-10	0.292765	1.67E-10
	CD3E	0.367829	4.04E-16	0.367829	4.04E-16
	CD2	0.383322	1.77E-17	0.383322	1.77E-17
**CD8+T cell**	CD8A	0.346485	2.30E-14	0.346485	2.30E-14
	CD8B	0.215771	3.16E-06	0.215771	3.16E-06
	CD86	0.686509	4.24E-65	0.686509	4.24E-65
	CSF1R	0.585082	2.04E-43	0.585082	2.04E-43
**TAM**	CCL2	0.710411	1.42E-71	0.710411	1.42E-71
	CD68	0.467805	2.78E-26	0.467805	2.78E-26
	IL10	0.512319	5.17E-32	0.512319	5.17E-32
**M1 macrophage**	IRF5	0.308985	1.37E-11	0.308985	1.37E-11
	PTGS2	0.289882	2.56E-10	0.289882	2.56E-10
	NOS2	-0.2026	1.25E-05	-0.2026	1.25E-05
**M2 macrophage**	CD163	0.679957	1.97E-63	0.679957	1.97E-63
	VSIG4	0.658727	2.56E-58	0.658727	2.56E-58
	MS4A4A	0.662905	2.72E-59	0.662905	2.72E-59
**Neutrophils**	CEACAM8	-0.18625	6.07E-05	-0.18625	6.07E-05
	ITGAM	0.670462	4.31E-61	0.670462	4.31E-61
	CCR7	0.311286	9.52E-12	0.311286	9.52E-12
**Natural killer cell**	KIR2DL1	0.196671	2.25E-05	0.196671	2.25E-05
	KIR2DL3	0.160361	0.000571	0.160361	0.000571
	KIR2DL4	0.19043	4.10E-05	0.19043	4.10E-05
	KIR3DL1	0.225887	1.04E-06	0.225887	1.04E-06
	KIR3DL2	0.207018	7.94E-06	0.207018	7.94E-06
	KIR3DL3	0.052956	0.258054	0.052956	0.258054
	KIR2DS4	0.168361	0.000296	0.168361	0.000296
**Dendritic cell**	HLA-DPB1	0.504539	5.98E-31	0.504539	5.98E-31
	HLA-DQB1	0.32912	4.95E-13	0.32912	4.95E-13
	HLA-DRA	0.507051	2.73E-31	0.507051	2.73E-31
	HLA-DPA1	0.513432	3.63E-32	0.513432	3.63E-32

## Discussion

The incidence of COAD has increased dramatically in recent years, and the heterogeneity of colorectal cancer makes it difficult to determine which patients require further treatment after surgical resection and which have a poor prognosis. Diagnostic and prognostic tools for the early detection and prediction of patient survival are limited. Many studies have focused on this question, and there have been many advances in uncovering the underlying mechanisms by which cancer occurs. Therefore, finding reliable diagnostic markers for COAD is still an important research focus.

The results showed that CTHRC1 expression in COAD tissues was higher than that in normal tissues, and the difference was statistically significant. This finding is consistent with previous studies, which also found that CTHRC1 protein is highly expressed in various types of cancer, such as lung, stomach, cervical, and breast cancers. Our analysis further confirmed this finding because we found that CTHRC1 was significantly overexpressed in most of the tumors in the TCGA data. These results suggest that CTHRC1 may be a diagnostic marker of multiple cancers. In addition, we found that CTHRC1 was associated with the clinical staging and histological subtypes of COAD. Previous studies have reported that CTHRC1 promotes tumor cell progression by affecting specific pathways in different cancer types. For example, CTHRC1 is elevated in cervical cancer and promotes metastasis via the Wnt/PCP pathway [21]. In contrast, CTHRC1 regulates the aggressiveness of NSCLC through the GSK-3β/β-catenin pathway [22]. Current studies have found that CTHRC1 is an intrinsic marker of COAD metastasis, and further revealed that CTHRC1 promotes COAD liver metastasis through TGF-β signaling remodeling infiltrated macrophages [23]. This suggested that CTHRC1 expression may be a convenient diagnostic biomarker for a variety of tumors, including COAD. In addition, CTHRC1 is highly expressed in COAD and associated with poor prognosis. This result is consistent with previous findings that high CTHRC1 expression is associated with poor COAD survival [24].

Gene mutations have important implications, including altering genetic content, disrupting genes, and causing phenotypic differences. We found that the main type of CTHRC1 change was mRNA upregulation, and copy number variation was the most common type which may be the reason for the high expression of CTHRC1 in COAD. Changes in the chromosomal structure of the CTHRC1 gene can lead to abnormal expression and dysfunction. In the current study, gene mutations in CTHRC1 accounted for 6% of colorectal adenocarcinomas. The gene mutation of CTHRC1 in COAD was 4.65%, and the change frequency of CTHRC1 in mucinous adenocarcinoma of the colon and rectum was the highest, up to 13.04%. These results suggest that high CTHRC1 mutation may increase the carcinogenesis of COAD. According to the GO analysis, we found that the functional network of CTHRC1 in COAD is involved in morphogenesis, endoplasmic reticulum lumen, extracellular matrix structural components, and collagen trimers, suggesting that CTHRC1 plays a biological role in morphogenesis and cytoskeletal tumorigenesis. These findings suggest that CTHRC1 may regulate transcription to influence cell biological function.

In addition, it is important to understand how changes in proteins that regulate normal transcription are involved in cancers. It has been reported that genomic instability may lead to the transformation of normal cells into a carcinogenic state, and protein kinases and their related signaling pathways will help stabilize and repair genomic DNA [25]. To further investigate the role of CTHRC1 in COAD, we used TCGA data for GSEA. The results showed that the high expression of CTHRC1 was enriched in various pathways and key biological functions, and was related to the occurrence of tumors, such as Gα signaling, GPCR ligand binding, neutrophil degranulation, interleukin signaling, and tumor-associated pathways. The mechanism of Gα classical signaling is to inhibit the cAMP-dependent pathway by inhibiting adenylate cyclase. The reduced production of cAMP in ATP results in decreased cAMP-dependent protein kinase activity, and mutations in Gα subunits lead to specific cancers [26]. Additionally, many cancer cells abnormally express GPCRs, including those from lung, prostate, colon, pancreas, and mesenchymal cancer cells, which stimulate cell proliferation, migration, invasion, and angiogenesis [27]. Among members of the GPCR family, gonadotropin-releasing hormone receptor (GnRH) has been reported to be overexpressed in various tumor cells such as melanoma, prostate and endometrial cancer, leiomyoma, breast cancer, choriocarcinoma, epithelial ovarian tumor, and stromal ovarian tumor [28]. Therefore, the GnRH receptor is a reliable target for the clinical treatment of cancer [29]. The molecular basis of neutrophil degranulation is not fully understood, although the SNARE and Rab proteins seem to play a central role [30]. The presence of neutrophil components in cytoplasmic granules is involved in tumor metastasis and angiogenesis and may be considered a biomarker for tumor prognosis [31]. The tumor microenvironment is an increasingly popular topic that may influence tumor progression and recurrence. Studies have shown that immune cells in the TME have protumor or antitumor activity [32]. They are considered to be important determinants of clinical outcome and immunotherapy response. We observed that CTHRC1 expression was closely related to COAD immune infiltration. For example, CTHRC1 expression was significantly positively correlated with multiple immune cells and immune cell markers. It is suggested that CTHRC1 may reflect not only the prognosis of the disease but also the immune status of the body. In conclusion, these results underlie the ability of CTHRC1 to potentially regulate immune cell recruitment and activation in COAD.

The present study improves our understanding of the relationship between CTHRC1 and COAD, although some limitations still exist. First, all the data analyzed in this study were retrieved from online databases, and further studies consisting of in vitro and in vivo experiments are required to validate our findings. Second, most of the analyses in the present study were performed based on mRNA levels of CTHRC1. A deeper analysis, based on protein levels, would make the data more convincing.

## Conclusion

This study systematically analyzed the relationship of CTHRC1 expression with COAD. The results showed that the expression of CTHRC1 is upregulated in COAD, and high CTHRC1 expression was correlated with clinical progression. In addition, Gα signaling, GPCR, neutrophil degranulation, and interleukin signal transduction may be the key pathways regulated by CTHRC1. The expression of CTHRC1 is closely related to the infiltration of various immune cells in COAD. Therefore, the biological function of CTHRC1 may play a vital role in the diagnosis and treatment of COAD.

## Data Availability

The data that support the findings of this study are openly available in TCGA database at https://portal.gdc.cancer.gov/.

## Abbreviations

BMP: Bone morphogenetic proteins
COAD: Colon adenocarcinoma
CTHRC1: Collagen triple helix repeat containing 1
DFS: Disease-free survival
GSEA: Gene set enrichment analysis
GO: Gene ontology
TGF-β: Transforming growth factor-β
TCGA: The Cancer Genome Atlas
